# Longitudinal population subgroups of CRP and risk of depression in the ALSPAC birth cohort

**DOI:** 10.1016/j.comppsych.2019.152143

**Published:** 2020-01

**Authors:** Emanuele F. Osimo, Jan Stochl, Stan Zammit, Glyn Lewis, Peter B. Jones, Golam M. Khandaker

**Affiliations:** a Department of Psychiatry, University of Cambridge, Cambridge, UK; b Cambridgeshire and Peterborough NHS Foundation Trust, Cambridge, UK; c Institute of Clinical Sciences, Imperial College London, London, UK; d Centre for Academic Mental Health, Population Health Sciences, Bristol Medical School, University of Bristol, Bristol, UK; e Division of Psychiatry, University College London, London, UK; f Department of Kinanthropology, Charles University, Prague, Czech Republic

**Keywords:** ALSPAC, CRP, C-reactive protein, Inflammation, Depression, Immunopsychiatry

## Abstract

**Background:**

Meta-analyses confirm increased circulating C-reactive protein (CRP) levels in depression. Longitudinal studies have linked one-off measurements of CRP at baseline with increased risk of developing depressive symptoms subsequently at follow-up, but studies with repeat CRP measures from the same individuals are scarce.

**Methods:**

We have examined whether longitudinal patterns of inflammation, based on three CRP measurements from childhood to early-adulthood, are associated with the risk of depression in early-adulthood in the Avon Longitudinal Study of Parents and Children, a prospective birth cohort.

**Results:**

Using Gaussian mixture modelling of available CRP data from age 9, 15 and 18 years, we identified four population clusters/sub-groups reflecting different longitudinal patterns of CRP: persistently low (N = 463, 29.5%); persistently high (N = 371, 24%); decreasing (N = 360, 23%); increasing (N = 367, 23.5%). The increasing group showed a steep increase in CRP levels between adolescence and early-adulthood. Participants in this group had a higher risk of moderate/severe depression at age 18 years, compared with those with persistently low CRP; adjusted odds ratio (OR) = 3.78 (95% Confidence Interval (CI), 1.46–9.81; p = 0.006). The odds of moderate/severe depression were also increased for the persistently high CRP group, but this was not statistically significant; OR = 2.54 (95% CI, 0.90–7.16).

**Limitations:**

Repeat CRP measures were available for a subset, who may not be representative of all cohort participants.

**Conclusions:**

The results suggest that an increasing pattern of inflammation from adolescence to early-adulthood is associated with risk of depression in early-adulthood.

## Highlights

• Using CRP data from age 9, 15 and 18 we identified four population subgroups.
• The subgroups represent persistently low, persistently high; decreasing; increasing CRP levels.
• Increasing CRP levels were associated with moderate/severe depression at age 18.

## 1 Introduction

Depression and other mood disorders are common in all populations [1], and a quarter of all cases emerge before 20 years of age [2,3]. Most current treatments for depression involve medication that affects the brain monoamine systems [4], however heterogeneity in clinical presentation and variability in treatment response suggest additional mechanisms are present. There is now ever increasing evidence for an association between depression and inflammation [[5], [6], [7], [8], [9], [10], [11], [12]]. CRP has been used most extensively as a measure of low-grade systemic inflammation in psychiatric [11,13,14] and physical conditions [15,16]. Meta-analysis of cross-sectional studies demonstrate that levels of CRP and inflammatory cytokines, such as IL-6, are elevated in acute depression [7,[17], [18], [19]]. Similarly, longitudinal studies suggest that increased levels of inflammatory markers in childhood/adolescence are associated with increased risks of depression and psychosis subsequently in adulthood [[20], [21], [22], [23], [24]]. Using prospective birth-cohort data, we reported that higher levels of IL-6 in childhood are associated with an increased risk of developing depression subsequently in early adulthood in a dose-dependent manner [22]. We also reported that higher IL-6 levels in childhood are associated with subsequent persistent depressive symptoms between the ages of 10 and 19 years [25]. Other studies have also found a longitudinal association between circulating IL-6 and CRP levels and risk of depression at follow-up [20,26]. Findings from longitudinal studies of childhood inflammatory markers suggest that inflammation precedes depression, so it could be a cause for depression rather than simply be a consequence of illness.

A feature of most existing epidemiological studies of depression is that they used a one-off measure of CRP to gauge inflammation. There is some evidence that CRP and IL-6 levels remain relatively stable over time [27,28], and studies have often excluded participants with suspected infection [22,29]. Nevertheless, repeated measures within individuals over time allow to detect the level and longitudinal pattern of inflammation more accurately. To our knowledge, not many studies have examined the association between depression and longitudinal pattern of inflammation during childhood, adolescence and young adulthood. We are aware of one population-based longitudinal study that measured CRP five times between age 9 and 21 years [30]. Despite repeated measures, CRP data from only one assessment was used as predictor, reporting no association with depression in adulthood.

We have investigated the longitudinal pattern of inflammation in the Avon Longitudinal Study of Parents and Children (ALSPAC), a UK general population birth cohort. We used data on serum CRP levels assessed at ages 9, 15 and 18 years to create population subgroups reflecting longitudinal pattern of CRP using cluster analysis, an unsupervised machine learning technique. We hypothesised that persistently high or increasing levels of CRP from childhood through to early-adulthood would be associated with the risk of depression in early-adulthood assessed at age 18 years.

## 2 Methods

### 2.1 Cohort description and sample

The ALSPAC is a general population-based birth cohort. Pregnant women resident in former county Avon, UK, with expected dates of delivery between 1st April 1991 and 31st December 1992 were invited to take part in the study. The initial number of pregnancies enrolled is 14,541 (for these at least one questionnaire has been returned or a “Children in Focus” clinic had been attended by 19/07/99). Of these initial pregnancies, there was a total of 14,676 foetuses, resulting in 14,062 live births and 13,988 children who were alive at 1 year of age.

Since age 7 years, the children attended annual clinical assessments during which they participated in various face-to-face interviews and physical tests. When the oldest children were approximately 7 years of age, an attempt was made to bolster the initial sample with eligible cases who had failed to join the study originally. As a result, when considering variables collected from the age of 7 onwards (and potentially abstracted from obstetric notes) there are data available for more than the 14,541 pregnancies mentioned above. The total sample size for analyses using any data collected after the age of 7 is therefore 15,247 pregnancies, resulting in 15,458 foetuses. Of this total sample of 15,656 foetuses, 14,973 were live births and 14,899 were alive at 1 year of age.

Detailed information about the ALSPAC cohort can be found on the study website (http://www.bristol.ac.uk/alspac), and the sample characteristics and methodology have been described previously [31,32]. Please note that the study website contains details of all the data that is available through a fully searchable data dictionary and variable search tool (http://www.bristol.ac.uk/alspac/researchers/our-data/).

The risk set for the current study includes 1627 cohort members who provided data on serum CRP levels at every follow-up at ages 9, 15 and 18 years. We excluded 66 participants who had CRP levels >10 mg/l (marker of suspected infection) at any point. Of the remaining risk set, 1462 participants took part in assessment for depression at age 18 years and were therefore included in the analyses.

Ethical approval for the study was obtained from ALSPAC Ethics and Law Committee and the Local Research Ethics Committees. Consent for biological samples has been collected in accordance with the Human Tissue Act (2004).

### 2.2 Measurement of CRP

At age 9, non-fasting blood samples were taken using standard procedures. At age 15, participants provided blood samples after a fast of at least 6 hours. Samples were immediately spun and frozen at −80 °C. There was no evidence of previous freeze–thaw cycles during storage. For high sensitivity CRP (hsCRP) an automated particle-enhanced immunoturbidimetric assay (Roche UK, Welwyn Garden City, UK) was used. All assay coefficients of variation were < 5%. At age 9, a valid measure of serum CRP was obtained from 5076 participants in total, which ranged from 0.01 to 67.44 mg/l (60 subjects over 10 mg/l). At age 15, a valid measure of serum CRP was obtained from 3490 participants in total, which ranged from 0.07 to 72.55 mg/l (60 subjects over 10 mg/l). Similar procedures were followed for measuring CRP at age 18. A valid measure of serum CRP was obtained from 3287 participants in total, which ranged from 0.02 to 176.1 mg/l (79 subjects over 10 mg/l).

### 2.3 Assessment of depression at 18 years

The computerised version of the Clinical Interview Schedule Revised (CIS-R) was self-administered by cohort participants in assessment clinics at average age 17.8 years (SD = 0.38). The CIS-R is a widely used, standardized tool for measuring common mental disorders in large community samples [33]. In the UK, CIS-R has been used in National Psychiatric Morbidity Survey, a household survey on 10,000 individuals representative of the UK population, in 1993 and 2007 [34,35]. The CIS-R is a fully structured assessment, suitable for trained social survey interviewers and does not require any expert knowledge on the part of the interviewers. As such, it can also be administered using personal computers in which the subjects self-complete the questionnaire [36].

The CIS-R elicits responses to symptoms of depression experienced in the past week, and provides a diagnosis of depression according to ICD-10 criteria [37]. ICD-10 criteria for mild, moderate and severe depression were used to categorise depression severity. These were used as outcome.

### 2.4 Measurement of other variables

We examined the association of CRP subgroups with sex, maternal education, father’s occupation, weight at birth, maternal Edinburgh Post-natal Depression Scale (EPDS) score, body mass index (BMI) at age 18, total IQ score at age 8, atopic disorder in participants, and rheumatism or arthritis in either parent. Sex was recorded at birth and was coded as a binary variable. Father’s occupation was recorded at birth according to the UK Office of National Statistics Classification System (Class I = professionals and higher managerial workers; II = intermediate occupations; IIIa = skilled non-manual occupations; IIIb = skilled manual occupations; IV = partly skilled occupations; V = unskilled occupations), and was coded as a categorical variable (non-manual, i.e. I + II + IIIa, v. manual occupations, i.e. IIIb + IV + V). Maternal depression was measured by EPDS at 18-, 32-week gestation, and 8-week post-partum. These three values were averaged and used as a continuous variable. BMI was recoded at age 18 years, which was calculated as weight in kilograms divided by height in metre squared. Full scale, verbal and performance IQ were measured by the Wechsler Intelligence Scale for Children, 3^rd^ UK edition (WISC III) (Wechsler et al. 1992). A shortened version of the test was applied by trained psychologists, whereby alternate items (always starting with item number 1 in the standard form) were used for all ten subtests with the exception of the coding subtest, which was administered in its standard form. Assessment of atopy was done through a postal questionnaire completed by parents at age 10 years old. Parents were asked whether anytime in the past a doctor had stated that the child suffered from asthma or eczema (original question: “Has a doctor ever actually said that your study child has asthma or eczema?”). Based on response to this question a single categorical exposure (or independent) variable was created, which included four groups: no asthma or eczema (reference group); only asthma or eczema; both asthma and eczema. Presence of a diagnosis of rheumatism or arthritis in either parent was also based on self-reported questionnaire data.

### 2.5 Statistical analysis

Statistical analyses and graph plotting were performed in R v3.4 [38].

#### 2.5.1 CRP variables preparation and population clustering

Due to skewed distributions, CRP data were log-transformed, and all subsequent analyses performed on log(CRP). Longitudinal population subgroups based on CRP data were created using Gaussian finite mixture modelling. Gaussian finite mixture modelling uses an expectation-maximisation algorithm; it is a ‘soft’ clustering algorithm which aims to find a small number of classes, homogeneous with respect to patterns in observed data (in our case, CRP levels over time). This is accomplished by decomposing complex distributions in multivariate spaces into a small number of Gaussian distributions. This procedure is implemented in R [38] within the mclust package [39]. This package has been shown to outperform similar packages [40]. Using this package, we fitted various models differing in the number of classes and cluster shapes. The evaluation of each model’s fit and the determination of the optimal number of classes were based on the Bayesian Information Criterion (BIC).

#### 2.5.2 Descriptive analysis

Descriptive analyses were performed using the psych v1.7 [41] package. The differences in prevalence between categorical variables were tested using χ^2^ tests on contingency tables, while differences in prevalence between continuous normally distributed variables were tested using ANOVA. Differences between several independent groups of non-normal continuous values were tested using a Kruskal-Wallis χ^2^ test.

#### 2.5.3 Calculation of odds ratios (OR) for depression

Multivariate multinomial log-linear models were used to calculate ORs and 95% confidence intervals (CIs) for depression associated with CRP subgroups. We included sex, maternal education, and BMI at 18 years as potential confounders, because these factors were associated with CRP sub-groups/clusters. Reference categories for the calculation of ORs were the persistently low group, male sex, lower maternal education (below A-level), and absence of depression. Analyses and plotting were performed using the packages nnet v7.3 [42], ggplot2 v2.2 [43], Cairo v1.5 [44], gridExtra v2.3 [45], dplyr v0.7 [46], and plyr v1.8 [47].

## 3 Results

### 3.1 Population subgroups of inflammation based on CRP data at ages 9, 15 and 18 years

**Fig. 1:**
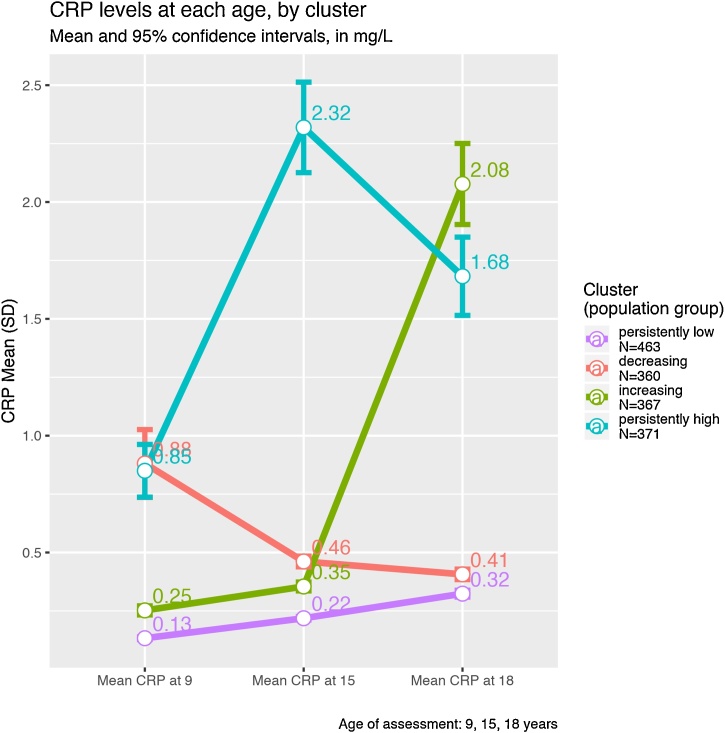
Mean (SD) CRP Levels at Ages 9, 15 and 18 Years in Four Population Subgroups of CRP in the ALSPAC Cohort.

Gaussian mixture modelling of the study population of 1561 participants with log(high-sensitivity CRP) values at 9, 15 and 18 years of age produced 2 potentially optimum models. Option 1 was an ellipsoidal clustering, with variable orientation, equal volume and shape, which identified 8 clusters in our data (BIC = -12631). Option 2 was also an ellipsoidal clustering, this time with variable orientation, volume and shape, which identified 4 clusters (BIC = -12634). Following the principle of maximal parsimony, and given that the BIC difference was minimal (3 points), we selected the second model as optimum. The four population sub-groups of CRP reflecting different patterns of inflammation over time were persistently low (N = 463, 29.5%); decreasing (N = 360, 23%); increasing (N = 367, 23.5%); and persistently high (N = 371, 24%). Subjects in the persistently low group showed the lowest average CRP values at all ages. Subjects in the decreasing group showed the second highest CRP values at 9 and 15 years of age, which decreased to the second lowest value at 18. Subjects in the increasing group showed the second lowest CRP values at 9 and 15 years, which increased to the highest levels at 18 years. Finally, subjects in the persistently high group showed the highest CRP values at 9 and 15 years, and the second highest at 18 (see Fig. 1, 3DFig. 1 : https://plot.ly/∼emosyne/2.embed, and Supplementary Table 1). For distributions of CRP at ages 9, 15 and 18 years, please see Supplementary results.

### 3.2 Association of CRP subgroups with sociodemographic and other factors

Increasing and persistently high patterns of CRP were associated with female sex, higher BMI at age 18, and lower maternal educational attainment; see Table 1.

**Table 1:** Characteristics of Population Sub-groups of CRP in the ALSPAC Cohort.

Characteristic	Total Sample	Population subgroup of CRP				Test statistic and p-value
		Persistently low group	Decreasing group	Increasing group	Persistently high group	
Sample, n (%)	1561 (100%)	463 (30%)	360 (23%)	367 (24%)	371 (24%)	
Male sex, n (%)	770 (49%)	250 (54%)	184 (51%)	160 (44%)	176 (47%)	χ^2^ = 9.8, df = 3, p = 0.02
Maternal education (A level and degree), n (%)	680 (46%)	181 (42%)	144 (42%)	173 (49%)	182 (51%)	χ^2^ = 9.8, df = 3, p = 0.02
Paternal social class (non-manual occupations), n (%)	422 (31%)	122 (31%)	81 (26%)	105 (31%)	114 (35%)	χ^2^ = 6.9, df = 3, p = 0.07
Birth weight, mean (SD), grams	3461.4 (530)	3503.2 (502)	3459.9 (546)	3407.9 (507)	3464.8 (565)	F = 2.1, df = 3, p = 0.1
Maternal depression, average EPDS score, median (IQR)	5.33 (5.67)	5.33 (5.67)	5.33 (5.67)	5.33 (5.00)	5.67 (5.33)	Kruskal-Wallis χ^2^ = 0.69, df = 3, p = 0.88
BMI at 18 years, mean (SD)	22.49 (3.59)	21.33 (2.57)	22.35 (3.37)	22.47 (3.51)	24.06 (4.32)	F = 42.35, df = 3, p < 0.0001
IQ at 8 years, mean (SD)	109.21 (16.33)	109.39 (16.25)	110.09 (16.15)	108.62 (16.34)	108.73 (16.60)	F = 0.597, df = 3, p = 0.62
Diagnosis of asthma or eczema, n (%)	386 (26.9%)	110 (25.8%)	93 (28.0%)	99 (28.4%)	84 (25.5%)	χ^2^ = 5.8, df = 6, p = 0.44
Diagnosis of rheumatic disease in parents, n (%)	53 (3.6%)	13 (3.0%)	15 (4.5%)	11 (3.1%)	14 (3.9%)	χ^2^ = 1.6, df = 3, p = 0.67
Arthritis in parents, n (%)	60 (4.0%)	14 (3.2%)	16 (4.7%)	19 (5.3%)	11 (3.1%)	χ^2^ = 3.5, df = 3, p = 0.31
Total CIS-R score at 18, median (IQR)	3 (7)	3 (7)	3 (6)	3 (7)	3 (6)	Kruskal-Wallis χ^2^ = 1.62, df = 3, p = 0.65
CIS-R total depression score at 18y, median (IQR)	2 (4)	1 (4)	2 (4)	1 (5)	2 (4)	Kruskal-Wallis χ^2^ = 1.24, df = 3, p = 0.74
ICD-10 depression at 18y, n (%)	96 (6.6%)	29 (6.7%)	15 (4.5%)	28 (8.1%)	24 (6.9%)	χ^2^ = 3.7, df = 3, p = 0.30
ICD-10 depression at 18 by severity:						
No depression, n (%)	1366 (93.4%)	404 (93.3%)	318 (95.5%)	318 (91.9%)	326 (93.1%)	χ^2^ = 11.1, df = 6, p = 0.08
Mild depression, n (%)	45 (3.1%)	19 (4.4%)	7 (2.1%)	9 (2.6%)	10 (2.9%)	
Moderate/severe, n (%)	51 (3.5%)	10 (2.3%)	8 (2.4%)	19 (5.5%)	14 (4.0%)	

### 3.3 Association between CRP subgroups and depression at 18 years

The prevalence of depression for individuals in the four CRP subgroups is presented in Table 1. Individuals in the increasing, compared with low, CRP group had a higher risk of ICD-10 diagnosis of depression at 18 years, but this was not statistically significant; adjusted OR 1.33 (95% CI, 0.73–2.39). Additional analysis based on depression severity showed that belonging to the increasing CRP group was associated with moderate/severe depression at 18 years; adjusted OR 3.78 (95% CI, 1.46–9.81); see Table 2. The odds of moderate/severe depression were also increased for the persistently high CRP group, compared with low CRP group, but this was not statistically significant; adjusted OR 2.54 (95% CI, 0.90–7.16).

**Table 2:** Association between Longitudinal Population Subgroups of CRP and Risk of Depression at Age 18 Years.

CRP Group	Total Sample	Any depression, No (%)	Odds Ratio (95% CI) for Depression	
			Unadjusted	Adjusted*
Persistently low	N = 433	29 (6.7%)	1 [reference]	1 [reference]
Decreasing	N = 333	15 (4.5%)	0.66 (0.35, 1.25)	0.80 (0.41, 1.59)
Increasing	N = 346	28 (8.1%)	1.23 (0.72, 2.10)	1.33 (0.73, 2.39)
Persistently high	N = 350	24 (6.9%)	1.03 (0.59, 1.80)	1.02 (0.54, 1.94)

CRP Group	Total Sample	Moderate/Severe depression, No (%)	Odds Ratio (95% CI) for Moderate/Severe depression	
			Unadjusted	Adjusted*
Persistently low	N = 433	10 (2.3%)	1 [reference]	1 [reference]
Decreasing	N = 333	8 (2.4%)	1.02 (0.40, 2.60)	1.83 (0.62, 5.41)
Increasing	N = 346	19 (5.5%)	2.41 (1.11, 5.26)	3.78 (1.46, 9.81)
Persistently high	N = 350	14 (4.0%)	1.73 (0.76, 3.96)	2.54 (0.90, 7.16)

* Adjusted for sex, maternal education, BMI at 18 years.

## 4 Discussion

Using population-based longitudinal birth-cohort data, we identified population sub-groups of young people characterised by different longitudinal patterns of peripheral inflammation levels. Subjects who showed a pattern of increasing CRP levels from childhood to early adulthood had a higher risk of moderate/severe depression at 18 years, compared with those who had persistently low CRP. Evidence for this association remained after controlling for sex, maternal education and BMI. Those with persistently elevated CRP also had increased odds of moderate/severe depression at 18, but this was not statistically significant. The results indicate that an increase in low-grade inflammation levels from childhood to early adulthood is strongly associated with risk of depression in early-adulthood.

The increasing group showed a nearly 6-fold increase in average CRP levels between the ages of 15 and 18 years, and showed a significant association with moderate/severe depression at 18 years. It is therefore possible that the strongest link between inflammation and depression lies in a recent increase, and that this is more important than a chronically high CRP level with regards to future risk of depression. Whether this recent CRP elevation could be a mediating factor for links between depression and past psychological stress, abuse/maltreatment is an important hypothesis that needs to be tested in further prospective samples.

Our findings add to increasing evidence supporting a link between the immune system, particularly low-grade systemic inflammation defined by elevated concentrations of circulating inflammatory markers, and depression and other major mental disorders [6,48,49]. CRP is an acute phase protein which has been studied extensively in depression and shown to be elevated in acutely unwell patients compared with controls in several meta-analyses [7,18,19]. Compounding cross-sectional evidence, recently longitudinal studies have shown that elevated cytokine levels precede, and so could potentially cause, depressive symptoms: elevated IL-6 or CRP levels in childhood are associated with an increased risk of developing depression and psychosis in adulthood [20,22,23,50]. We are now more confident than ever to suggest a causal role for inflammation in depression, as two recent studies have used Mendelian randomisation to further investigate the matter. In [51], the authors find that a genotype variant associated with decreased CRP levels is also associated with reduced odds of depression. In [52], the authors found that a poly-genic risk score for high CRP levels was significantly associated with increases in the risk of depression. These studies suggest that, if the genes linked with high CRP are linked with an increase in the risk of depression, it is unlikely that this association is only due to a true causal link rather than reverse causation or confounding. This study adds to the existing evidence by showing that increasing CRP levels between adolescence and young-adulthood are associated with a subsequent ICD-10 diagnosis of moderate/severe depression in young-adulthood at age 18 years. This finding fits nicely with a causal role of high CRP in depression, as it means that depression is most likely to manifest when CRP levels rise close to the index time of examination.

Based on repeat measure of CRP we report that individuals with increasing levels of inflammation over time, especially during adolescence, have higher risk of moderate/severe depression in early-adulthood. In this study we did not examine potential reasons for the increases in CRP levels during adolescence, but this is likely to include both genetic and environmental factors. Adolescence is a time of profound physical and psychological transformation. This is also a stressful time for many individuals due to, for instance, forming new relationships, peer pressure, bullying. Increasing independence and experimentation during this developmental epoch is also associated with fast-food consumption, smoking, drinking, substance use - all of these factors could increase levels of inflammation. In future, studies are required to test why a sub-group of adolescents experience a sharp increase in their level of inflammation.

### 4.1 Strengths

The use of a general population birth cohort, a relatively large sample, and particularly repeated prospective assessment of an inflammatory marker are some of the strengths of this study. The exclusion of participants with CRP levels of >10 mg/l at any point minimised potential confounding by chronic inflammatory conditions or current infection. In addition, we adjusted regression models for maternal education (a marker of socio-economic status), sex and BMI, which are linked with inflammation levels.

### 4.2 Limitations

A key limitation is missing data. Of 14,062 live births in ALSPAC, 1627 cohort members provided serum CRP levels at all time points of 9, 15 and 18 years of age. Out of these, we excluded 66 participants who, at any point, had CRP levels >10 mg/l, and of these, 1462 participants took part in psychiatric assessment at age 18 years and were therefore included in this study. Thus, the group with CRP data at all three time points that formed the basis for our analysis may not be representative of all ALSPAC participants. Attrition in prospective cohort studies including ALSPAC is associated with lower socio-economic status and increased mental illness. Selective attrition of participants more likely to have the outcome of interest (depression) may have increased the possibility of null findings in our sample. Due to a limited sample size for repeat CRP data, we did not include additional confounders such as childhood trauma, smoking, poor diet, low levels of physical activity, and sleep disturbance, as this would have reduced the sample size further, leading to decreased statistical power.

### 4.3 Conclusions

In summary, using repeated measurements of CRP from childhood to young-adulthood, we report that an increasing pattern of inflammation from adolescence to early-adulthood is associated with risk of moderate/severe depression in early-adulthood. Future studies should investigate the causes of this increase in inflammatory markers in the second decade of life in previously non-inflamed individuals, and the relationship these causes may have with depression.

## Declaration of Competing Interest

The authors declare that the research was conducted in the absence of any commercial or financial relationships that could be construed as a potential conflict of interest.
