# Supplementary material for: Longitudinal population subgroups of CRP and risk of depression in the ALSPAC birth cohort
Source: Compr Psychiatry. 2020 Jan;96:152143. doi: 10.1016/j.comppsych.2019.152143 (PMC6945112; doi:10.1016/j.comppsych.2019.152143)
Supplement: Supplementary file 1 [file mmc1.docx]

Osimo et al, Longitudinal Population Subgroups of CRP and Risk of Depression in the ALSPAC birth cohort

Supplementary materials

# **Supplementary results**

logCRP values followed a pseudo-normal distribution; logCRP values at 15 years showed a systematic shift to the left (Supplementary Figure 1).

# **Supplementary figures**

**Supplementary Figure 1: log(CRP) Distributions by Age**


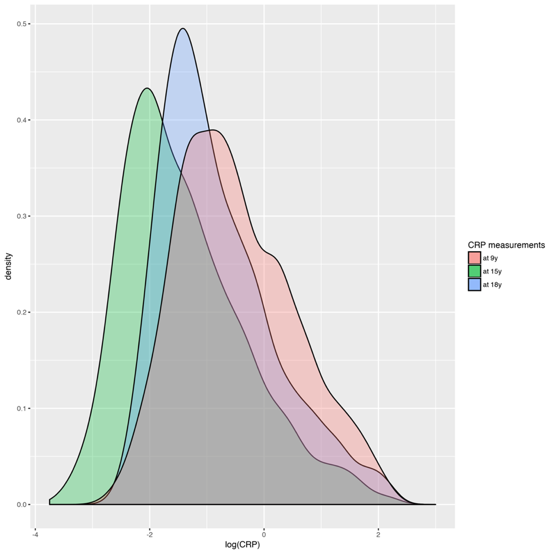


# **Supplementary Tables:**

**Supplementary Table 1: Mean (SD) loh(CRP) Levels at Ages 9, 15 and 18 Years in Four Population Subgroups in the ALSPAC Cohort**

| Population subgroup | N | log(CRP) Levels, mean (SD), mg/L | | | |
| --- | --- | --- | --- | --- | --- |
|  |  | 9 years | 15 years | 18 years | All-age |
| Persistently low | 463 | -2.13 (0.49) | -1.58 (0.37) | -1.26 (0.52) | -1.66 (0.35) |
| Decreasing | 360 | -0.90 (1.24) | -0.90 (0.53) | -1.04 (0.54) | -0.95 (0.51) |
| Increasing | 367 | -1.64 (0.76) | -1.18 (0.54) | 0.49 (0.67) | -0.78 (0.43) |
| Persistently high | 371 | -0.78 (1.12) | 0.57 (0.73) | 0.07 (1.00) | -0.05 (0.57) |
| Overall | 1561 | -1.41 (1.09) | -0.82 (0.98) | -0.48 (1.02) | -0.9 (1.1) |
